# Knowledge, attitude and practice of Lebanese parents towards childhood overweight/obesity: the role of parent-physician communication

**DOI:** 10.1186/s12887-022-03279-1

**Published:** 2022-04-14

**Authors:** Helene Barbe Zoghby, Elsa Sfeir, Marwan Akel, Diana Malaeb, Sahar Obeid, Souheil Hallit

**Affiliations:** 1 Faculty of Medicine, University of Bourgogne, Dijon, France; 2 School of Medicine and Medical Sciences, Holy Spirit University of Kaslik, P.O. Box 446, Jounieh, Lebanon; 3 School of Pharmacy, Lebanese International University, Beirut, Lebanon; 4 School of Pharmacy, Gulf Medical University, Ajman, United Arab Emirates; 5 Social and Education Sciences Department, School of Arts and Sciences, Lebanese American University, Jbeil, Lebanon; 6 Research Department, Psychiatric Hospital of the Cross, Jal Eddib, Lebanon; 7 Psychology Department, College of Humanities, Effat University, Jeddah, 21478 Saudi Arabia

**Keywords:** Knowledge, Attitude, Practice, Obesity, Parent-physician communication, Children

## Abstract

**Background:**

Childhood overweight and obesity is one of the most challenging public health problems facing both developed and developing countries. Several studies carried worldwide assessed predisposing risk factors for childhood obesity, however, few addressed the Middle East region and particularly Lebanon. We aimed in our study to assess factors associated with knowledge, attitude and practice of Lebanese parents regarding childhood overweight, particularly the parent-physician communication.

**Methods:**

This cross-sectional study conducted between September and December 2019. The questionnaire used was the standardized questionnaire of “Parent Questionnaire Curriculum” from the “We Can program” (Ways to Enhance Children’s Activity and Nutrition).

**Results:**

A higher parent-physician communication score (Beta = 0.05) was significantly associated with higher knowledge, whereas having a higher intermediate vs low income (Beta = -0.65) was significantly associated with lower knowledge. A higher knowledge global score (Beta = 1.25), a higher parent-physician communication score (Beta = 0.18), and having a university education level compared to illiterate/primary/complementary (Beta = 2.59) were significantly associated with better attitude. A better attitude (Beta = 0.92) and a higher global knowledge score (Beta = 0.6) were significantly associated with a better practice.

**Conclusion:**

This study found that a better parent-physician communication and higher knowledge were associated with better attitude and practice towards obesity. Given the rise in childhood obesity worldwide, identifying factors that help reduce childhood obesity, is becoming mandatory. Our results can open up to future studies addressing strategies to prevent childhood obesity in Lebanon.

**Supplementary Information:**

The online version contains supplementary material available at 10.1186/s12887-022-03279-1.

## Background

Childhood overweight is a serious problem that places children and adolescents at risk of poor physical and psychological health. It is defined as a body mass index between the 85^th^ and the 95^th^ percentile of growth curves charts [1]. Childhood overweight/obesity is one of the biggest challenges in the twenty-first century because of its rapid increase worldwide [2]. A study conducted in 2010 showed that globally, the number of overweight children was estimated to be over 35 million in developing countries and 42 million in developed countries [3]. In terms of childhood overweight, the Middle East is the third most affected region after the USA and Europe. A study conducted in 2014, showed that 38.4% of Lebanese children aged between 6 and 19 years are overweight and 13.2% are obese [4], higher than the worldwide prevalence estimated at 9.1% in 2020 [5].

Children’s overweight results from an interaction between genetic, environmental, behavioral, and social factors. Lebanese children are at risk of consuming large amounts of unhealthy foods, while spending three or more hours a day in front of the screen [6, 7]. In addition, they are largely exposed to food advertisements in which 80% of the products presented do not meet nutritional standards recommended by the World Health Organization due to inefficient media regulation [4].

Parental behaviors have been previously described as a factor influencing childhood obesity affecting directly or indirectly the knowledge and attitude of the society [8]. The majority of parents of overweight children are unaware of their children’s weight and the potential risks of this condition on their health [9]. On another hand, parents who perceive their children as overweight are more likely to encourage their children to be more physically active and restrict unhealthy food intake [10]. In addition, parent-focused interventions have shown good consequences in reducing child overweight in comparison to child-focused ones [11]. Furthermore, the parental level of education was shown to be a major contributing factor to children’s overweight, however, it played a controversial role in children obesity; higher parental education was associated with lower [12] vs higher levels of childhood obesity [13, 14]. The association between socioeconomic status and childhood obesity is also controversial; in high-income countries, childhood obesity was reported to be higher in families of low socioeconomic class, while in low-income countries, obesity was lower in families with a low socioeconomic class [14, 15].

Overweight children were shown to be at higher risk for developing non-communicable diseases (diabetes and cardiovascular disorders) related to obesity in adulthood [16]. Overweight can also have a negative impact on children’s psychological health [16]. Overweight children had lower self-esteem, poor academic performance and a low quality of life [17]. Hence, prevention of childhood overweight seems essential. Consequently, implementing preventive strategies should be based on conscientious investigations of childhood obesity determinants and predisposing risk factors.

Several studies carried worldwide assessed predisposing risk factors for childhood obesity, however few addressed the Middle East region, particularly Lebanon [18]. None addressed parental knowledge, attitude and parent-physician communication towards childhood obesity. We aimed in our study to assess factors associated with knowledge, attitude and practice of Lebanese parents regarding childhood overweight/obesity, particularly the parent-physician communication.

## Methods

### Study design

This cross-sectional study was conducted between September and December 2019 and enrolled participants from community pharmacies from all Lebanese districts (Beirut, Bekaa, Mount Lebanon, South Lebanon and North Lebanon). Pharmacies were chosen conveniently from each governorate. All adults entering the pharmacy were approached; those who were Lebanese and a parent of a child between two and eleven years were eligible for study enrollment. Parents with more than one child in this age range had to fill one survey for each child. Parents were free to accept or refuse to participate, with no financial compensation offered for participants. Participants who met the inclusion criteria were briefed about the topic, and the different aspects of the questionnaire before filling it, while being assured of the anonymity of the response, by one of the collaborators of this research project.

### Minimal sample size

The Epi-info software estimated the minimal sample size needed at 384, based on a 5% error and a 50% percentage of good knowledge of parents about obesity (the 50% value was chosen since it will yield the highest sample size in the absence of similar studies in the country). A total of 393 (78.6%) questionnaires out of 500 distributed was collected back and analyzed.

### Translation procedure

Two certified translators performed the forward and back translation (from English to Arabic then to English). The principal investigator verified this translation and matched the two English versions to detect inconsistencies and solve discrepancies. Minor corrections were done and solved by consensus between all involved parties.

### Questionnaire

The questionnaire was composed of five sections: the first one collects socio-demographic information (age, gender, education level and household monthly income). The following three sections gathered information regarding knowledge, attitude and practice (KAP) about alimentation, physical activity, and time spent in front of screens. The last one collected information regarding the level of parent-physician communication. The scale used was composed of 15 items, scored on a four-point Likert scale, with higher scores indicating a better parent-physician communication [19].

Knowledge, attitudes and practice global scores have been calculated separately. Concerning knowledge questions, a score of 1 was assigned for every right answer, and 0 for a wrong answer or a non-response. The total knowledge score was out of 20.

We have used a Likert scale with 1 to 5 scores (1 = strongly disagree, 5 = strongly agree) for attitude and practice propositions. The attitude and practice global scores were out 95 and 135 respectively. The three scores were divided later on into low, intermediate and high scores using the visual binning option in SPSS.

The questionnaire used in our study was the standardized questionnaire of “Parent Questionnaire Curriculum” from the “We Can program” (Ways to Enhance Children’s Activity and Nutrition) [20, 21], which is an updated version of Birsch and al (2004) “Child Feeding Questionnaire”. “We Can” is a youth obesity-prevention initiative providing activities and programs, with the aim of improving nutritional choices, increasing physical activity and reducing screen time in youth. It was prepared for the National Heart, Lung, and Blood Institute National Institutes of Health and is composed of four programs. One of them is Energize our Families: Curriculum for Parents and Caregivers, for the education of parents. The Parent Questionnaire Curriculum evaluates the level of KAP about nutrition, physical activity and screening time of children’s parents, before and after an educative intervention.

We also added the following questions about restriction, affective feeding, pressure to eat food and parents’ model, extracted from the Child Feeding Questionnaire de Birsh and al [22]: “I intentionally keep some food out of my child’s read”, “I offer sweets (candy, ice-cream, cake or pastries) as a reward for good behavior”, “My child should always eat all of the food on her plate”, “I have to be especially careful to make sure my child eats enough”, “If my child says I’m not hungry, I try to get him to eat anyway”. Questions were scored on a five-point Likert scale (1 = strongly agree, 5 = strongly disagree).

### Statistical analysis

Data was analyzed using the SPSS (Statistical Package for the Social Sciences) software v.25. Missing data represented less than 10% of the total database and therefore was not replaced. The knowledge, attitude, and practice scores were normally distributed, as verified by a skewness and kurtosis values between -2 and + 2 [23]. These assumptions were consolidated by the sample size exceeding 300 [24]. The Student t and ANOVA tests were used to compare two and three or more means. Pearson test was used to correlate two continuous variables. Three linear regressions were conducted taking the knowledge, attitude, and practice scores as dependent variables respectively. Variables that showed a *p* < 0.2 in the bivariate analysis were considered as independent ones to minimize the risk for confounding variables effect [25]. Significance was set at *p* < 0.05.

## Results

The reliability analysis of the scales used in this study were very good: knowledge global scale (α = 0.7), attitude (α = 0.834), practice (α = 0.846) and parent-physician communication (α = 0.946). The sociodemographic characteristics of the parents are summarized in Table 1. The mean age of the parents was 33.50 ± 7.17 (89% females).

**Table 1:** Sociodemographic characteristics of the participants (*N* = 393)

Variable	N (%)
**Gender**	
Male	43 (11.0%)
Female	348 (89.0%)
**Education level**	
Illiterate/primary/complementary	51 (13.3%)
Secondary	127 (33.1%)
University	206 (53.6%)
**Governorate**	
Beirut	153 (38.9%)
Mount Lebanon	112 (28.5%)
North Lebanon	13 (3.3%)
South Lebanon	25(6.4%)
Bekaa	90 (22.9%)
**Living area**	
Rural	137 (35.6%)
Urban	248 (64.4%)
**Household monthly income**	
Low (< 1000 USD)	78 (20.2%)
Intermediate (1000–2000 USD)	174 (45.1%)
High (> 2000 USD)	134 (34.7%)
	**Mean ± SD**
**Age (in years)**	33.50 ± 7.17

^*^Numbers might not add up to the total number because of missing values

### Description of the different knowledge, attitude and practice scores

The description of the knowledge, attitude and practice global scores are summarized in Table 2. When using the visual binning option to divide the scores into three categories, the results showed that 170 (43.3%) of the participants had low knowledge (scores ≤ 13), whereas 113 (28.8%) and 110 (28.0%) had moderate (scores between 14 and 15) and good (scores ≥ 16) knowledge respectively. Moreover, 134 (34.1%) of the participants had poor attitude (scores ≤ 69), whereas 128 (32.6%) and 131 (33.3%) had moderate (scores between 70 and 76) and good (scores ≥ 77) attitude respectively. Finally, 138 (35.1%) of the participants had poor practice (scores ≤ 86), whereas 140 (35.6%) and 115 (29.3%) had moderate (scores between 87 and 98) and good (scores ≥ 99) practice respectively.

**Table 2:** Description of the different knowledge, attitude and practice scores

Score	Mean	Median	Standard deviation	Minimum	Maximum
Knowledge global score	13.67	14	2.90	1	19
Attitude global score	71.87	73	9.94	17	95
Practice global score	90.05	92	13.80	31	117
Parent-physician communication	77.90	78	13.96	20	100

### Bivariate analysis of factors associated with the knowledge, attitude and practice scores

Higher mean knowledge, attitude and practice global scores were found in those with a university level of education compared to the other categories, in those living in Mount Lebanon compared to the other governorates and in those with a high household monthly income compared to the other categories (Supplementary Table 1).

Higher global knowledge score was significantly associated with higher global attitude and practice scores, as well as a better parent-physician communication score. Moreover, a better global attitude score was significantly associated with better practice and parent-physician communication scores, whereas a higher practice score was significantly associated with better parent-physician communication score (Supplementary Table 2). More details about the correlations between the subscales scores can be found in Supplementary Table 3.

### Multivariable analysis

The results of a first linear regression, taking the global knowledge score as the dependent variable and the sociodemographic characteristics and the parent-physician communication score as independent variables, showed that a higher patient-communication score (Beta = 0.05) and living in Mount Lebanon vs Beirut (Beta = 1.04) were significantly associated with higher knowledge, whereas having a higher intermediate vs low income (Beta = -0.65) was significantly associated with lower knowledge (Table 3, Model 1).

**Table 3:** Multivariable analysis

**Model 1: Linear regression taking the global knowledge score as the dependent variable**					
**Variable**	**Unstandardized Beta**	**Standardized Beta**	***p*-value**	**95% Confidence Interval**	
Parent-physician communication	0.05	0.26	< 0.001	0.03	0.07
Mount Lebanon vs Beirut^a^	1.04	0.16	0.001	0.42	1.65
Intermediate vs low^a^ income	-0.65	-0.11	0.019	-1.20	-0.11
**Model 2: Linear regression taking the attitude score as the dependent variable**					
**Variable**	**Unstandardized Beta**	**Standardized Beta**	***p*-value**	**95% Confidence Interval**	
Knowledge global score	1.25	0.37	< 0.001	0.95	1.55
Parent-physician communication	0.18	0.25	< 0.001	0.11	0.24
University education level vs illiterate/primary/ complementary	2.59	0.14	0.002	0.93	4.25
**Model 3: Linear regression taking the practice score as the dependent variable**					
**Variable**	**Unstandardized Beta**	**Standardized Beta**	***p*-value**	**95% Confidence Interval**	
Attitude global score	0.92	0.66	< 0.001	0.81	1.02
Knowledge global score	0.60	0.13	0.001	0.25	0.94
Mount Lebanon vs Beirut^a^	4.50	0.15	< 0.001	2.39	6.62
Bekaa vs Beirut^a^	2.29	0.07	0.042	0.09	4.49
Parent-physician communication	0.04	0.04	0.319	0.05	0.82

^a^Reference group

Variables entered in model 1: Parent-physician communication, Education level, governorate, household monthly income

Variables entered in model 2: Parent-physician communication, Education level, governorate, and global knowledge score

Variables entered in model 3: Parent-physician communication, Education level, governorate, global knowledge score, global attitude score, household monthly income and house crowding index

A second linear regression, taking the global attitude score as the dependent variable, showed that a higher knowledge global score (Beta = 1.25), a higher patient-communication score (Beta = 0.18), and having a university education level compared to illiterate/primary/complementary (Beta = 2.59) were significantly associated with better attitude (Table 3, Model 2).

A third linear regression, taking the global practice score as the dependent variable, showed that better attitude (Beta = 0.92), a higher global knowledge score (Beta = 0.6), living in Mount Lebanon (Beta = 4.50) and Bekaa (Beta = 2.29) compared to Beirut were significantly associated with a better practice (Table 3, Model 3).

## Discussion

We found in our study that a higher parent-physician communication was associated with better knowledge, and attitude toward childhood obesity. In addition, better knowledge and attitude were associated with better practice. Those results are in line with previous findings [26] showing that an improvement in the parent-physician communication can result in a better weight management in children. Nowadays, the relationship between doctors and parents have shifted to be based on communication that leads to a shared decision-making [27, 28]. Consequently, patient-care provider communication can lead to a better knowledge and a higher adherence to treatment recommendation, with better medical outcomes [29]. Furthermore, better health outcome and compliance to obesity treatment were reported when the pediatric patient is implicated in the communication [26]. When addressing childhood obesity, fewer than 25% of parents of obese children have been previously informed that their child was overweight [30, 31]. In fact, a study conducted on obese Latin children showed that there was a lack of direct communication of predisposing factors for obesity [31]. The American academy of pediatrics recommended the implementation of communication strategies for information and obesity plan reduction, given that better communication will lead to better knowledge and attitude, and consequently better global practice [32]. As better knowledge and attitude were linked to better practice in our study, we can probably say that the lack of knowledge about childhood obesity can be partially responsible for the increasing numbers of childhood overweight. Those results were different from those found in a study conducted in Malaysia where better knowledge was not associated with better attitude and practice [33].

Parental education was associated with better attitude in our study. Those results were similar to previous findings showing that higher parental education level was associated with lower childhood overweight [34], attributed to the better attitude toward childhood overweight prevention. Highly educated parents are aware of the benefit of breastfeeding, for example, in preventing childhood obesity and overweight and tend to breastfeed their infants [35]. In addition, highly educated parents are more aware of the healthy lifestyle, acquire better dieting skills during their education and can, consequently, implement those dietary habits in their family in a better way [36]. Finally, the difference in social norms between high and low educated parents can explain the difference between attitude toward childhood obesity and overweight preventions [34]. However it is important to mention that previous studies, including one study conducted in Lebanon, showed that higher maternal education was associated with higher childhood overweight and obesity [14, 37]. This was explained by the fact that the increasing number of working, high educated mothers in Lebanon led to an increase in appointing paid helpers at home. Consequently, mothers tend to pay less attention to their children’s behaviors and could be responsible for an increase in overweight/obesity by two folds [14].

Finally, we found in our study that parental knowledge was lower in parents with intermediate monthly income in comparison with those with a low income. Parental nutritional knowledge was found to be an important contributor for childhood obesity [38]since it influences parental food shopping and preparation and food delivery to their children. Previous studies [34, 39, 40] have showed, in opposition to what we found in our study, that parents with lower income were associated with lower knowledge about obesity because they tend to have a worse understanding of nutritional habits and knowledge [38, 41]. However, this association is not valid in Lebanon given the economic crises Lebanese people are facing.

### Clinical implications

Our results should encourage Lebanese pediatricians and healthcare workers to enhance the parent-physician communication. In addition, this study can raise awareness on the importance of increasing knowledge toward obesity among parents and children, in order to get better attitude and practice. The role of awareness campaigns in schools for health promotion that educates about child’s wellbeing, including healthy eating and physical activity, is a key factor for the success in the fight against childhood overweight and obesity.

### Limitations

This study is a cross-sectional study that did not take into account different behaviors over a period of time; the results were collected through a self-administered questionnaire, thus, subject to an information bias. A selection bias is present since parents were recruited from community pharmacies and because of the refusal rate for participation. A residual confounding bias is also possible since not all factors associated with KAP are considered in this paper. The questionnaire used was not adapted to the Lebanese population. The cutoff points used for the scales were arbitrary. No standardization was made for the Lebanese population. In addition, the number of male participants was very low compared to females, and the representation of the different Lebanese governorates was not homogenous; thus, the generalizability of the results and their extrapolation to the wider community may need more studies to support our results.

## Conclusion

Children worldwide, and particularly Lebanese children, are at a high risk for childhood obesity. Consequently, raising awareness about predisposing and protective risk factors, seems mandatory. This study highlighted the importance of educating parents, and improving parent-physician communication in achieving better knowledge, attitude and practice towards obesity. Those results may serve as a first step for the implementation of educational programs and strategies to reduce childhood obesity.

## Supplementary Information

**Additional file 1: Supplementary Table 1. **Bivariate analysis of factors associated with the knowledge, attitudeand practice global scores. **Supplementary Table 2. **Bivariate analysis of continuous variables associated with the knowledge, attitude and practice global scores. **Supplementary Table 3. **Bivariate analysis of continuous variables associated with the subscales scores.

## Data Availability

All data generated or analyzed during this study are not publicly available to maintain the privacy of the individuals’ identities. The dataset supporting the conclusions is available upon request to the corresponding author.

## Abbreviations

KAP: Knowledge, attitude and practice
SPSS: Statistical Package for the Social Sciences
