# Supplementary material for: Knowledge, attitude and practice of Lebanese parents towards childhood overweight/obesity: the role of parent-physician communication
Source: BMC Pediatr. 2022 Apr 14;22:209. doi: 10.1186/s12887-022-03279-1 (PMC9009025; doi:10.1186/s12887-022-03279-1)
Supplement: Supplementary file 1 — Additional file 1: Supplementary Table 1. Bivariate analysis of factors associated with the knowledge, attitudeand practice global scores. Supplementary Table 2. Bivariate analysis of continuous variables associated with the knowledge, attitude and practice global scores. Supplementary Table 3. Bivariate analysis of continuous variables associated with the subscales scores. [file 12887_2022_3279_MOESM1_ESM.docx]

| **Supplementary Table 1. Bivariate analysis of factors associated with the knowledge, attitude and practice global scores.** | | | |
| --- | --- | --- | --- |
| **Variable** | **Knowledge global score** | **Attitude global score** | **Practice score** |
| **Gender** |  |  |  |
| Male | 13.74 ± 3.39 | 70.35 ± 9.65 | 90.91 ± 12.97 |
| Female | 13.71 ± 2.77 | 72.28 ± 9.46 | 90.18 ± 13.51 |
| p-value | 0.515 | 0.213 | 0.788 |
| **Education level** |  |  |  |
| Illiterate/primary/complementary | 13.17 ± 3.37 | 70.00 ± 9.89 | 86.20 ± 13.55 |
| Secondary | 13.05 ± 2.69 | 68.82 ± 10.76 | 87.38 ± 13.74 |
| University | 14.13 ± 2.83 | 74.24 ± 8.83 | 92.64 ± 13.11 |
| p-value | **0.001** | **<0.001** | **<0.001** |
| **Governorate** |  |  |  |
| Beirut | 13.29 ± 2.64 | 71.41 ± 10.64 | 87.48 ± 14.65 |
| Mount Lebanon | 14.66 ± 2.67 | 75.14 ± 7.23 | 96.52 ± 8.81 |
| North Lebanon | 12.92 ± 3.64 | 70.54 ± 10.42 | 86.38 ± 16.06 |
| South Lebanon | 12.76 ± 3.79 | 71.20 ± 15.58 | 85.36 ± 20.23 |
| Bekaa | 13.44 ± 2.97 | 68.94 ± 8.58 | 88.18 ± 12.42 |
| p-value | **<0.001** | **<0.001** | **<0.001** |
| **Living area** |  |  |  |
| Rural | 13.51 ± 3.02 | 71.03 ± 10.79 | 89.64 ± 14.10 |
| Urban | 13.73 ± 2.85 | 72.23 ± 9.34 | 90.16 ± 13.31 |
| p-value | 0.713 | 0.233 | 0.778 |
| **Household monthly income** |  |  |  |
| Low (<1000 USD) | 13.78 ± 2.56 | 72.51 ± 7.71 | 91.49 ± 11.95 |
| Intermediate (1000-2000 USD) | 13.29 ± 2.73 | 70.88 ± 9.56 | 88.34 ± 13.28 |
| High (>2000 USD) | 14.21 ± 3.09 | 73.33 ± 10.21 | 92.40 ± 13.70 |
| p-value | **0.005** | 0.103 | **0.021** |

**Post hoc analysis**: knowledge score: education level (secondary vs university p=0.023); Governorate (Beirut vs Mount Lebanon p<0.001; Mount Lebanon vs South p=0.026; Mount Lebanon vs Bekaa p=0.02); household monthly income (intermediate vs high p=0.045)

Attitude score: education level (Illiterate/primary/complementary vs university p=0.016; secondary vs university p<0.001); Governorate (Beirut vs Mount Lebanon p=0.022; Mount Lebanon vs Bekaa p<0.001)

Practice score: education level (Illiterate/primary/complementary vs university p=0.007; secondary vs university p=0.002); Governorate (Beirut vs Mount Lebanon p<0.001; Mount Lebanon vs South p=0.002; Mount Lebanon vs Bekaa p<0.001); household monthly income (intermediate vs high p=0.023)

| **Supplementary Table 2. Bivariate analysis of continuous variables associated with the knowledge, attitude and practice global scores.** | | | |
| --- | --- | --- | --- |
| **Variable** | **Knowledge global score** | **Attitude global score** | **Practice score** |
| Knowledge global score | 1 |  |  |
| Attitude global score | 0.410^a^ | 1 |  |
| Practice global score | 0.432^a^ | 0.696^a^ | 1 |
| Parent-physician communication | 0.252^a^ | 0.394^a^ | 0.345^a^ |
| House crowding index | 0.049 | 0.002 | 0.157^b^ |
| Age | 0.077 | -0.032 | 0.038 |

| **Supplementary Table 3. Bivariate analysis of continuous variables associated with the subscales scores.** | | | | | | | | | | | | | | |
| --- | --- | --- | --- | --- | --- | --- | --- | --- | --- | --- | --- | --- | --- | --- |
|  | Food Knowledge | Physical activity knowledge | Sedentary Knowledge | Food attitude | Physical activity attitude | Sedentary attitude | Food practice | Physical activity practice | Sedentary practice | Model parent | Restrictive parent | Pressure parent | Coming to physician for obesity |  |
| Food Knowledge | 1 |  |  |  |  |  |  |  |  |  |  |  |  |  |
| Physical activity knowledge | 0.239^a^ | 1 |  |  |  |  |  |  |  |  |  |  |  |  |
| Sedentary Knowledge | 0.259^a^ | 0.162^b^ | 1 |  |  |  |  |  |  |  |  |  |  |  |
| Food attitude | 0.189^a^ | 0.272^a^ | 0.157^b^ | 1 |  |  |  |  |  |  |  |  |  |  |
| Physical activity attitude | 0.153^b^ | 0.270^a^ | 0.109^c^ | 0.333^a^ | 1 |  |  |  |  |  |  |  |  |  |
| Sedentary attitude | 0.242^a^ | 0.330^a^ | 0.261^a^ | 0.381^a^ | 0.368^a^ | 1 |  |  |  |  |  |  |  |  |
| Food practice | 0.234^a^ | 0.394^a^ | 0.156^b^ | 0.537^a^ | 0.339^a^ | 0.473^a^ | 1 |  |  |  |  |  |  |  |
| Physical activity practice | 0.115^c^ | 0.361^a^ | 0.113^c^ | 0.321^a^ | 0.610^a^ | 0.393^a^ | 0.466^a^ | 1 |  |  |  |  |  |  |
| Sedentary practice | 0.154^b^ | 0.271^a^ | 0.162^b^ | 0.375^a^ | 0.343^a^ | 0.736^a^ | 0.519^a^ | 0.458^a^ | 1 |  |  |  |  |  |
| Model parent | 0.212^a^ | 0.421^a^ | 0.220^a^ | 0.462^a^ | 0.466^a^ | 0.532^a^ | 0.712^a^ | 0.751^a^ | 0.651^a^ | 1 |  |  |  |  |
| Restrictive parent | 0.112^c^ | 0.267^a^ | 0.017 | 0.458^a^ | 0.330^a^ | 0.326^a^ | 0.740^a^ | 0.409^a^ | 0.356^a^ | 0.491^a^ | 1 |  |  |  |
| Pressure parent | 0.070 | 0.196^a^ | 0.075 | 0.277^a^ | 0.135^b^ | 0.212^a^ | 0.107^c^ | 0.220^a^ | 0.215^a^ | 0.318^a^ | 0.338^a^ | 1 |  |  |
| Coming to physician for obesity | 0.069 | 0.124^c^ | 0.105^c^ | 0.282^a^ | 0.202^a^ | 0.289^a^ | 0.245^a^ | 0.209^a^ | 0.325^a^ | 0.259^a^ | 0.234^a^ | 0.104^c^ | 1 |  |

^a^p<0.001; ^b^p<0.01; ^c^p<0.05
